# GDF9 is Transiently Expressed in Oocytes before Follicle Formation in the Human Fetal Ovary and is Regulated by a Novel NOBOX Transcript

**DOI:** 10.1371/journal.pone.0119819

**Published:** 2015-03-19

**Authors:** Rosemary A. L. Bayne, Hazel L. Kinnell, Shiona M. Coutts, Jing He, Andrew J. Childs, Richard A. Anderson

**Affiliations:** 1 MRC Centre for Reproductive Health, University of Edinburgh, Queen’s Medical Research Institute, Edinburgh, United Kingdom; 2 Department of Comparative Biomedical Sciences, The Royal Veterinary College, London, United Kingdom; Colorado State University, UNITED STATES

## Abstract

During human fetal ovary development, the process of primordial follicle formation is immediately preceded by a highly dynamic period of germ cell and somatic cell reorganisation. This is regulated by germ-cell specific transcription regulators, by the conserved RNA binding proteins DAZL and BOLL and by secreted growth factors of the TGFβ family, including activin βA: these all show changing patterns of expression preceding follicle formation. In mice, the transcription factor Nobox is essential for follicle formation and oocyte survival, and NOBOX regulates the expression of GDF9 in humans. We have therefore characterised the expression of GDF9 in relation to these known key factors during follicle formation in the human fetal ovary. mRNA levels of *GDF9*, *BMP15* and *NOBOX* were quantified by qRT-PCR and showed dramatic increases across gestation. GDF9 protein expression was localised by immunohistochemistry to the same population of germ cells as those expressing activin βA prior to follicle formation but did not co-localise with either BOLL or DAZL. A novel *NOBOX* isoform was identified in fetal ovary that was shown to be capable of up-regulating the GDF9 promoter in reporter assays. Thus, during oogenesis in humans, oocytes go through a dynamic and very sharply demarcated sequence of changes in expression of these various proteins, even within individual germ cell nests, likely to be of major functional significance in determining selective germ cell survival at this key stage in ovarian development. Transcriptional variation may contribute to the range of age of onset of POI in women with *NOBOX* mutations.

## Introduction

The timely breakdown of oocyte nests into individual primordial follicles in the immature ovary is critical to female fertility, and occurs in fetal life in the human. Although a number of factors important for this process have been identified in recent years (reviewed in [1]), the picture is incomplete, and there are also differences between species in both the timing and synchronicity of follicle formation and in the role of signalling molecules.

Following sex determination, human ovarian development is characterised by a radial pattern of germ cell differentiation [2,3]. Thus less mature, mitotic germ cells (which express characteristic pluripotency markers such as OCT4 and LIN28 [4]) are found in the peripheral zone of the ovary, with the formation then breakdown of germ cell nests (also termed germ cell cysts [5]) in progressively deeper layers towards the central medulla of the ovary. The onset of meiosis [6,7] follows germ cell nest formation and germ cells switch from expressing the RNA binding protein DAZL, which is required for entry into meiosis [8], reviewed in [9], to expressing the related protein BOLL as cells go through zygotene and pachytene [10]. Meiotic arrest at the diplotene stage of prophase 1 occurs as primordial follicles form from approximately 17 weeks gestation. Follicle formation is associated with a switch back to DAZL expression [10].

Members of the TGFβ family of growth factors are important throughout ovarian development and function [11]. Activin A is expressed by germ cells immediately prior to follicle formation [12,13] and may have a functional role in that process by regulating kit ligand expression in adjacent somatic cells [14]. Growth and differentiation factor 9 (GDF9) is essential for oocyte-dependent development of ovarian follicles beyond the primary follicle stage [15], and *Gdf9* transcripts are also present in murine germ cell nests and in primordial follicles from embryonic day 19.5 (E19.5) [16]. In the hamster ovary, where expression of GDF9 has been observed before follicle formation [17], GDF9 and siRNAs against *GDF9* were able to promote or inhibit respectively primordial follicle formation [17,18]. Such species differences in GDF9 activity may in part be related to a single amino acid change in the receptor binding region in non-rodents which determines whether GDF9 is secreted in an active or a latent form [19,20]. BMP15 (also known as GDF9B), is co-expressed with GDF9 in oocytes and also shows species differences in its function: loss of *BMP15* in sheep leads to sterility [21] while in mice its loss has a mild effect on fertility [22].

It appears that while TGFβ family growth factors including activin and potentially GDF9, are key factors regulating germ cell development, oocyte specific transcription factors such as NOBOX act as master regulators of these and other key oocyte genes [23,24]. Mutation of *NOBOX* in women leads to primary ovarian insufficiency (POI) and may account for a substantial number of such cases compared to other single gene mutations [25,26]. Deletion of *Nobox* in mice [16] leads to increased numbers of both oocyte nests and primordial follicles at post natal day 3 (PND3) compared to wild-type littermates but few primary and no secondary follicles were present and oocytes were subsequently lost so that none remained by 6 weeks after birth. In *Nobox-^/-^* mouse ovaries, abnormal cell-cell adhesion was identified by electron microscopy [27], with failure of somatic pre-granulosa cells to encase individual oocytes as oocyte cyst breakdown proceeds in the process of primordial follicle formation—this leads to only partially enclosed oocytes which die during early postnatal life. A number of genes are down-regulated in the ovaries of *Nobox^-/-^* mice, including *Gdf9* [16,28,29,30]. NOBOX binds to the promoter regions of genes it regulates through a conserved NOBOX Binding Element (NBE) [29] which has the sequence TA(A/G)TT(G/A). Thus the primary defect in *Nobox* ^-/-^ ovaries appears to occur during follicle formation and may involve loss of GDF9 signalling. NBEs have been identified in the mouse [29] and human [25] promoter regions of *GDF9* and these have been demonstrated to bind NOBOX *in vitro* suggesting that NOBOX may control *GDF9* expression directly. In this respect, in a recent study of patients with POI [31], one patient was shown to have a tandem duplication of a 479bp fragment in the *GDF9* promoter containing 3 NBEs and an E-box suggesting increased sensitivity to NOBOX.

Existing data on GDF9 [32] and BMP15 [33] expression in human fetal ovary are limited to stages beyond 21 weeks when many follicles have already formed. *NOBOX* transcript levels have been reported to increase during fetal ovarian development [34,35] but have not been studied in detail. There are no previous data on the expression of GDF9 in the human fetal ovary prior to follicle formation, or of its potential interactions with NOBOX in regulating follicle formation. We have investigated here whether GDF9 and BMP15 are expressed in the human fetal ovary at the time of oocyte nest breakdown and primordial follicle formation and whether *GDF9* expression at this time might be regulated by NOBOX. We have determined the structure of *NOBOX* transcripts in the fetal ovary, identified a novel transcript and examined whether expression of the protein thus encoded can activate the proximal NBE in the human *GDF9* promoter *in vitro*. Our data indicate that human germ cells transiently express both activin βA and GDF9, with the latter likely to be under NOBOX control, in the lead-up to primordial follicle formation.

## Materials and Methods

### Ethics Statement

Ethical approval for this study was obtained from Lothian Research Ethics Committee (study code LREC 08/S1101/1). All participants gave informed written consent in accordance with national guidelines.

### Tissue

Human fetal ovaries (gestations 8–20 weeks) were obtained following medical termination of pregnancy as described previously [12]. Written informed consent was obtained and the study was approved by the Lothian Research Ethics Committee. Ovaries were removed and snap frozen and stored at -80°C for later RNA extraction, or fixed in Bouins fluid or 4% Normal Buffered Formalin (NBF) prior to wax embedding for immunohistochemistry. A total of 28 fetal ovary specimens were used in this study. Sections of adult marmoset, fixed in Bouins, were obtained from historical stocks maintained in the Centre for Reproductive Health, University of Edinburgh.

### RNA Extraction, Quantitative RT-PCR analysis

RNA was extracted from fetal ovaries using the RNeasy Mini Kit (14 weeks gestation onwards) or RNeasy Micro Kit (8–12 weeks gestation; both Qiagen, Crawley, UK) with on-column DNase I digestion. First strand cDNA was synthesised from 500ng RNA using Superscript Vilo Reverse Transcriptase Master Mix (Life Technologies, Paisley, UK).

Quantitative reverse transcriptase-PCR (qRT-PCR) was performed for human *GDF9*, *BMP15*, *NOBOX* and *RPL32* using 500nM each of the primer pairs described in Table 1 and Brilliant III SYBR Green Master Mix (Agilent Technologies, Wokingham, UK) on the ABI7900 Fast system with SDS2.4 software (Life Technologies, Paisley, UK). Standard curves for products of each gene transcript were used for quantitative comparisons relative to *RPL32* which does not change between first and second trimester gonads. Melt curves were analysed to confirm specific products. Data were analysed by the Kruskal-Wallis test and Dunn’s Multiple Comparisons post-hoc test using GraphPad Prism 6.0 software.

**Table 1:** Primers used for qRT-PCR and to determine expressed NOBOX exons.

	Sequence	Product Size
GDF9_F1	TAGTCAGCTGAAGTGGGACA	277bp
GDF9_R1	ACGACAGGTGCACTTTGTAG	
BMP15_F1	GGCTCCTAGGGCATTCACTG	196bp
BMP15_R1	CCTCGGTTTGGTCTGAGAGG	
NOBOX_F1	GACCCTTTCCCTCAGGAGTC	210bp
NOBOX_R1	CATCAGCAGTGGCATCAGTT	
RPL32_F	CATCTCCTTCTCGGCATCA	152bp
RPL32_R	AACCCTGTTGTCAATGCCTC	
		**Reference**
NOBOX_2Fa	CCCCAACATGATCCCTTAGA	[42]
NOBOX_3Rb	CAGTTCCTCACTCTGAGTGT	[42]
NOBOX_3Fc	CACCATCTCAGGAGAGAAGA	[42]
NOBOX_4F	CTGGAGGAGCTAGAGAAGAT	[42]
NOBOX_6R	AAAGGTATCCAGAGGGGACT	[42]
NOBOX_7R	AAGTCTGGTCAGAAGTCAGC	[42]
		**GenBank/ ENSEMBL Reference**
NOBOX_1Fd	ATGGCTCTCCTTTTGACACT	NM_001080413.3
NOBOX_1Fe	CCTGGCTGTACCTGAATTTCC	NM_001080413.3
NOBOX_2Rb	GGACTGTTCAGGTATCTCT	NM_001080413.3
NOBOX_2Fc	ATGGAACCCACAGAGAATCC	ENST00000223140
NOBOX_7F	GCTGACTTCTGACCAGACTT	NM_001080413.3 / ENST00000223140
NOBOX_8R	CTATATTCCCAGCAGGTGGTTG	NM_001080413.3 / ENST00000223140
NOBOX_9R	CTAGGGGACATGGCTATTCTT	NM_001080413.3 / ENST00000223140

### Immuno-localisation of GDF9 in human fetal ovary

Paraffin-embedded ovaries were cut into 5μm sections, with immunohistochemistry performed as previously described [36]. Affinity purified goat polyclonal anti-GDF9 antibody raised against recombinant mouse GDF9 (AF739; R&D Systems Europe Ltd, Abingdon, UK) was diluted 1:200 for DAB staining, with bound antibody detection using the ImmPRESS HRP anti-Goat IgG (peroxidase) Polymer Detection Kit (MP7405; Vector Laboratories, Peterborough, UK) and DAB (Dako, Glostrup, Denmark). Negative controls were incubated with normal goat IgG (sc_2028; Santa Cruz Biotechnology Inc., Heidelberg, Germany) at equivalent concentrations, in place of primary antiserum. For double and triple immunostaining of GDF9 with activin βA or DAZL and BOLL, methods were as previously described [10]. Monoclonal anti-activin βA antibody raised against amino acids 82–114 (E4, 1:200; a gift from NP Groome) was visualised with biotinylated goat anti-mouse antibody (1:500; BA9200, Vector Laboratories Ltd, Peterborough, UK) and Streptavidin-Alexa 546 (Molecular Probes, Leiden, The Netherlands). Anti-GDF9 antibody (1:50; AF739; R&D Systems) was detected with fluorescent tyramide with TOPRO counterstain. For triple fluorescent immunohistochemistry, monoclonal mouse anti-DAZL antibody raised against the C terminal domain of human DAZL (1:400; MCA2336, AbD Serotec, Kidlington, UK) and monoclonal mouse anti-BOULE (BOLL) antibody raised against amino acids 185–284 of human BOLL(1:200; Ab57696, Abcam, Cambridge, UK) were detected sequentially with peroxidase conjugated chicken anti-mouse antibody (sc-2692; Santa—Cruz Biotechnology Inc., Heidelberg, Germany) followed by tyramide Cy5 and Cy3 respectively (PerkinElmer, Bucks, UK). Anti-GDF9 antibody (1;200) was detected using fluorescein-labelled tyramide detection via a peroxidase conjugated chicken anti-goat antibody (sc-2691; Santa—Cruz Biotechnology). Counterstain was DAPI.

The proportion of GDF9, DAZL and BOLL expressing cells across the ovary was determined by analysis of 1422 germ cells in sections of 19–20 fetal ovary triple stained for GDF9, DAZL and BOLL. Nuclear diameters of immuno-stained germ cells were measured using Image J software on a total of 18 images collected from 3 different specimens of 19–20 weeks gestation. Data were analysed by ANOVA with Dunn’s multiple comparison post hoc test using Graphpad Prism 6.0 software.

### Determination of the structure of the human NOBOX gene by RT-PCR

RT+ and RT- first strand cDNA from 19 and 20 week fetal ovaries was PCR amplified with a range of *NOBOX* exon-specific primers (Table 1) in order to determine the structure of the expressed *NOBOX* gene. Each reaction was then electrophoresed on 1.8% TAE agarose gels alongside a 100bp DNA size ladder (Promega, Southampton,UK) and compared to predicted sizes depending on exon/intron structure.

### Transient transfection of HEK293 cells and Luciferase/ β-galactosidase Reporter Assays

The human GDF9 promoter-luciferase plasmid pGL3-hGDF9 [25] was a kind gift from N. Binart. ZP2 (pA3luc-ZP2) and ZP3 (pA3luc-ZP3) promoter-luciferase constructs were prepared by PCR-amplifying 528bp and 1.4kb fragments respectively of proximal human ZP2 and ZP3 promoters from genomic DNA with primers containing *XhoI* and *HindIII* restriction sites and cloned into the vector pA3LucPL (a derivative of pA3Luc [37] which has a polylinker inserted into the cloning site upstream of the luciferase reporter gene). Plasmid DNA for transfection was prepared using Macherey-Nagel NucleoBond Xtra Maxi Plus kits (Fisher Scientific). HEK293 cells [38] (ATCC CRL-1573, obtained from a colleague after a small number of passages) were transfected using Lipofectamine 2000 with OptiMEM as diluent (both Life Technologies). Individual transfections contained 500ng of pGL3-hGDF9, pA3luc-ZP3 or pA3luc-ZP2, with the addition of 50ng pCMV6-NOBOX or pCMV6-Entry vector, with transfecting DNA made up to a total of 1μg using pcDNA3. 10ng of a β-galactosidase reporter plasmid was added to each to act as an internal control for transfection efficiency. Transfections were incubated for 48 hours at 37°C in growth medium (Minimal Essential Medium + GlutaMAX + 10% Fetal Bovine Serum, all Life Technologies). Promoter activity was assayed using a Tropix Dual Light Luciferase Assay kit (Life Technologies) with sequential detection of both luciferase and β-galactosidase activity in the same sample. In all experiments, transfections and luciferase/β-galactosidase assays were each performed in duplicate and experiments were repeated at least 5 times. Data were analysed by ratio-paired t-tests on log transformed data using GraphPad Prism 6.0 software.

## Results

### Expression of GDF9, BMP15 and NOBOX mRNAs across gestation

**Fig 1:**
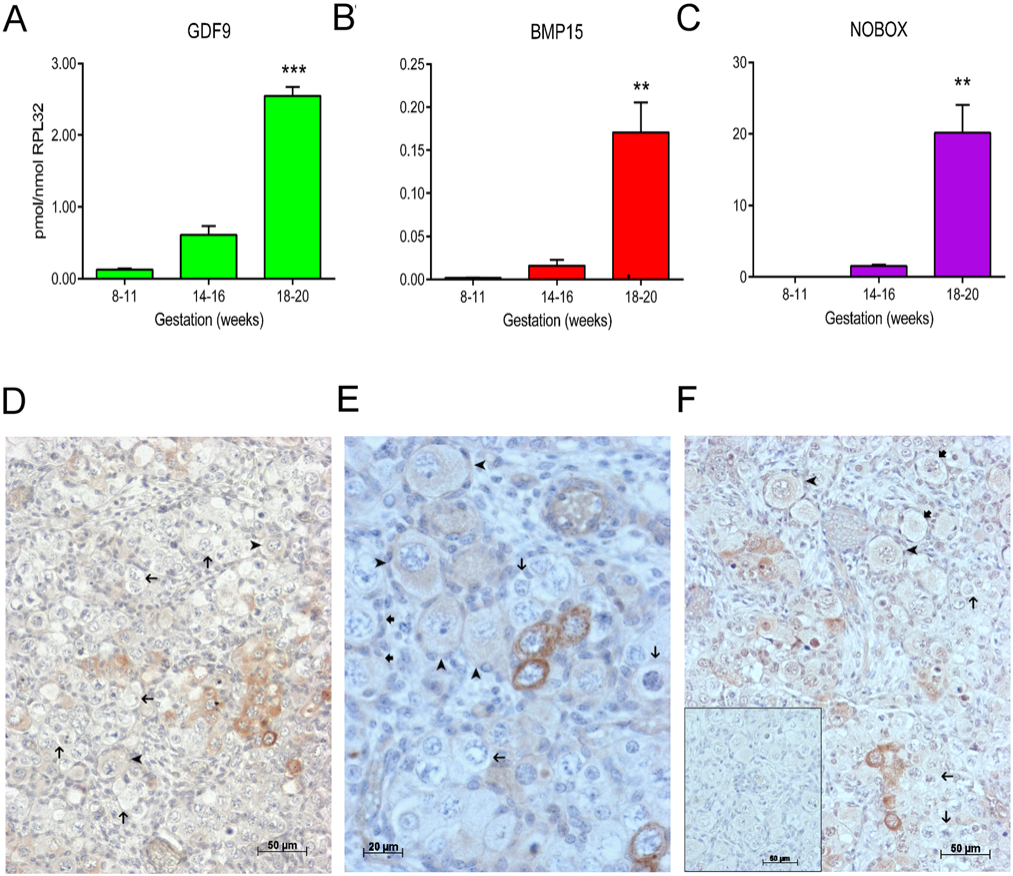
GDF9 is expressed in the human fetal ovary. qRT-PCR analysis of *GDF9* (A), *BMP15* (B) and *NOBOX* (C) mRNA expression in human fetal ovary across the gestational range of 8 to 20 weeks. Ovaries (n = 5–7 per group) were grouped according to developmental stage and transcript levels measured relative to those of *RPL32*. Bars indicate mean±sem. Statistically different levels are indicated by asterisks above the columns, thus expression of *GDF9* at 18–20 weeks was significantly higher than at 8–11 weeks (p<0.005) as was expression of *BMP15* and of NOBOX (both p<0.01). DAB immunohistochemical detection of GDF9: 19 week (D, E) and 20 week (F) human fetal ovary stained with anti-GDF9 antibody or normal goat IgG negative control (F inset)—positive staining is brown. Thick arrows indicate primordial follicles and thin arrows germ cells that are not stained for GDF9 while the arrowheads indicate primordial follicles that are positive for GDF9. Scale bars are 50μm (D and F) and 20μm (E).

Expression was investigated on samples (n = 5–7 per group) representing key stages of human ovarian development, *ie* 8–11 weeks gestation (oogonial proliferation), 14–16 weeks (ongoing proliferation and entry into meiosis) and 18–20 weeks (germ cell nest breakdown and primordial follicle formation). Expression of *GDF9* at 8–11 weeks was low, increased some 4-fold at 14–16 weeks and a further 4-fold in 18–20 week specimens (p<0.005, Fig. 1A). Similarly, *BMP15* was virtually undetectable at 8–11 weeks, increased about 10-fold at 14–16 weeks and a further 10–fold at 18–20 weeks (p<0.01, Fig. 1B). The pattern of *NOBOX* expression was also very similar with very low levels in 8–11 week ovaries, a small increase at 14–16 weeks and an approximately 8-fold increase at 18–20 weeks (p<0.01, Fig. 1C). Thus, *NOBOX*, *GDF9* and *BMP15* show marked increases in expression coincident with the initiation of oocyte nest breakdown and primordial follicle formation in the human fetal ovary.

### GDF9 is localised in a subset of germ cells prior to follicle formation in the human fetal ovary

DAB staining demonstrated expression of GDF9 in the cytoplasm of small clusters of germ cells at later gestations of human fetal ovary (19–20 weeks; Fig. 1D-F), with only a small number of GDF9 positive germ cells detectable from 16+ weeks and without clear expression at earlier gestations (S1A,B Fig.). Even at later gestations, the great majority of germ cells did not express GDF9 (Fig. 1D, examples indicated by thin arrows), and those enclosed in primordial follicles (Fig. 1E, F) in the fetal ovary either showed reduced or no expression (arrowheads and thick arrows respectively) compared to positive cells still in oocyte clusters. No staining was observed in normal IgG negative controls (Fig. 1F, inset and S1E Fig.) or in fetal testis (S1C Fig.; arrows indicate examples of germ cells in the tubules) but specificity was confirmed by clear staining of oocytes within follicles in adult marmoset ovary although, as in human fetal ovary, primordial follicles stained more weakly than larger follicles (S1D Fig.).

Previous immunohistochemistry studies [33,39] for human BMP15 have produced somewhat conflicting results, perhaps in part due to different tissue fixation methods and different antibodies, but there is very little information about BMP15 expression in fetal ovary before 21 weeks of gestation. We investigated two BMP15 (GDF9B) antibodies: neither of them (mouse anti-GDF9B—mAb28A [40]) and rabbit anti-GDF9B; Santa Cruz sc-28911) was able to detect BMP15 in either fetal ovaries or in follicles of NBF or Bouins fixed adult human or marmoset ovaries.

### Expression of GDF9 matches that of Activin βA in human fetal ovary

**Fig 2:**
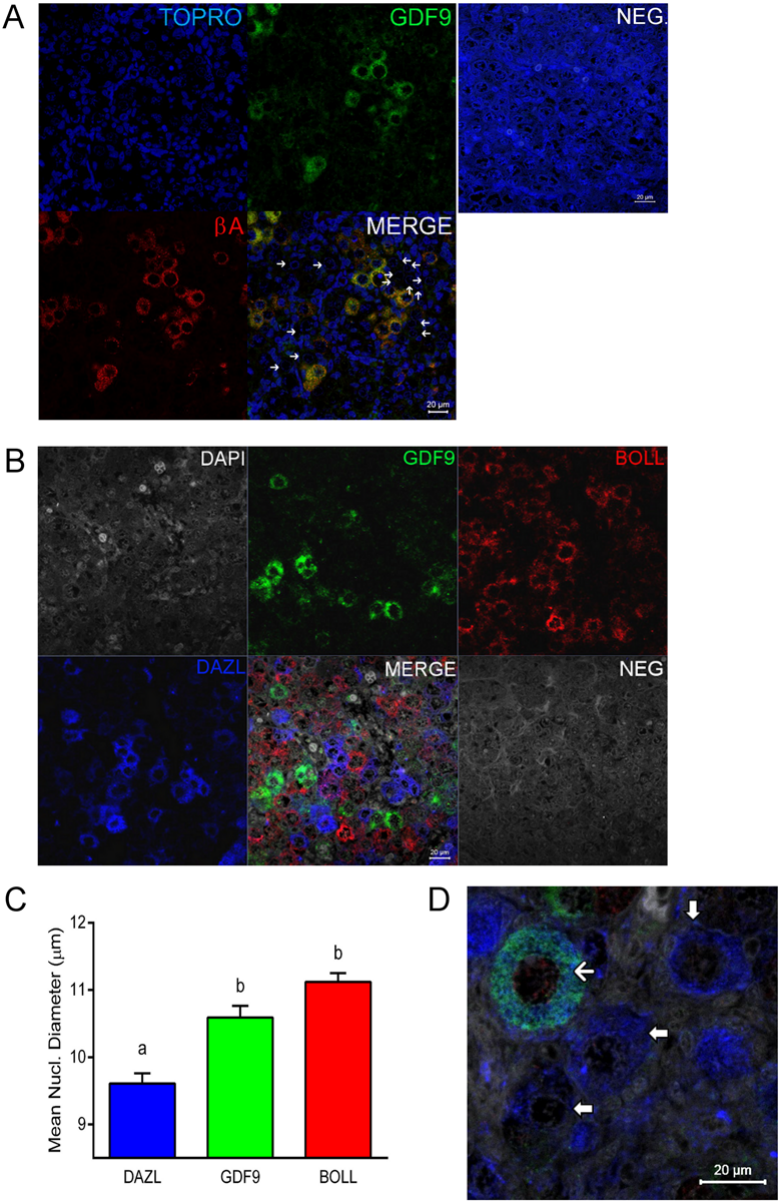
Co-localisation of GDF9 with activin βA but not DAZL or BOLL prior to follicle formation. (A) Double immunohistochemistry of 18 week fetal ovary stained for GDF9 (green) and activin βA (red), thus in the merged image co-expression is yellow. Unstained germ cells are indicated with arrows. Counterstain is TOPRO. (B) Triple fluorescent immunohistochemistry for GDF9 (green), DAZL (blue) and BOLL (red) in 20 week human fetal ovary with DAPI as counterstain (grey). Split channel and merged images in (A) and (B) are shown as are merged images of non-immune serum negative control (NEG). Scale bars are 20μm. (C) Nuclear diameters of DAZL, BOLL and GDF9 stained germ cells indicates that GDF9 positive cells are significantly larger (p<0.001) than DAZL but not BOLL expressing cells (bars indicate mean ± sem). (D) Higher magnification merged image of GDF9/DAZL/BOLL immunohistochemistry showing one large primordial follicle is positive for both GDF9 and DAZL but other follicles are positive only for DAZL.

The pattern of expression of GDF9 was similar to that of activin βA [13]. Double immunohistochemistry (Fig. 2A) demonstrated that essentially all GDF9-expressing oocytes (green) also expressed activin βA (red). Thus, activin βA and GDF9 are both transiently and simultaneously expressed in human fetal oocytes, but expression of both is switched off before or at primordial follicle formation.

### GDF9 expressing oocytes are distinct from those expressing DAZL or BOLL

In order to assess the developmental stage of oocytes expressing GDF9, we performed triple immunohistochemistry (Fig. 2B) with antibodies against the RNA binding proteins DAZL and BOLL, which show marked changes at these stages of ovarian development, with DAZL expressed in oogonia and oocytes in early meiotic prophase 1 while those in later stages up to late pachytene express BOLL [10]. There was no overlap between DAZL or BOLL and GDF9 expressing germ cells prior to follicle formation although oocytes expressing each of these were adjacent to each other, within the same oocyte nest (Fig. 2B) indicating marked non-synchrony. GDF9 positive cells were larger (10.6±0.2 μm, n = 73) than those expressing DAZL (9.6±0.2 μm, n = 165; p<0.001) but not significantly different from those expressing BOLL (11.1±1.6 μm, n = 141; Fig. 2C). GDF9 staining oocytes represented some 19.2% of the total number of oocytes within GDF9/DAZL/BOLL positive clusters but the overall proportion of GDF9, DAZL and BOLL expressing cells determined by analysis of 1422 germ cells across the ovary at 19–20 weeks gestation demonstrated that GDF9 was expressed by 6% of germ cells, compared to 69% for DAZL and 20% for BOLL, with some 5% of germ cells immuno-negative for all 3. In the 20 week ovary, positive DAZL (blue) staining of primordial follicles (Fig. 2D) confirmed our previous observation [10] that this protein is switched back on again once follicles have formed but most follicles were negative for GDF9 (green) with only a few larger primordial follicles (possibly in the earliest stages of growth activation) expressing both DAZL and GDF9 (Fig. 2D).

### Structure of the human NOBOX gene

**Fig 3:**
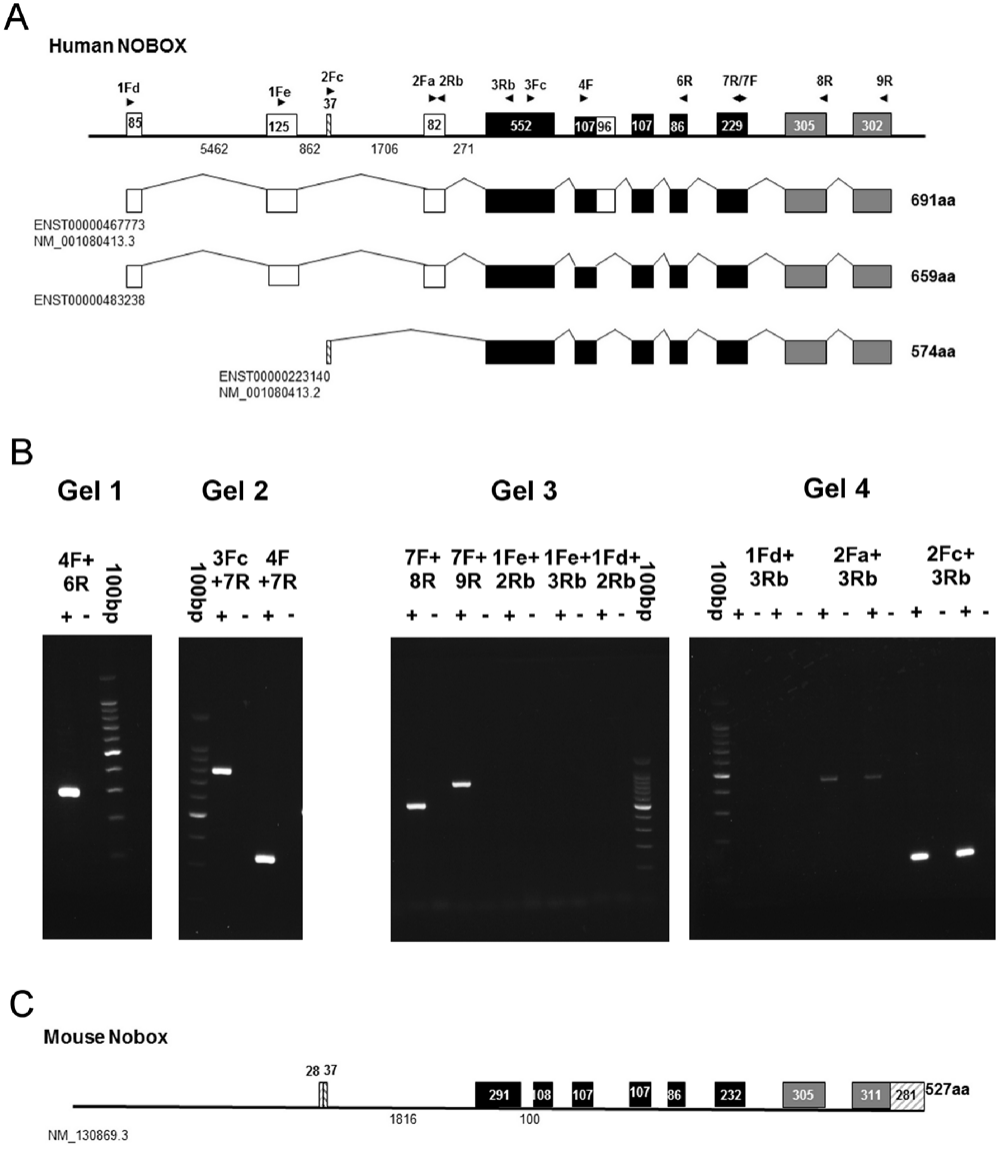
Structure of the human *NOBOX* gene and expression of exons in the human fetal ovary. (A) Database analysis of human *NOBOX* transcripts identified 3 possible transcripts for human NOBOX. Only those exons marked in black have been confirmed previously at the experimental level [41]. Exon and intron sizes are indicated and primers used for RT-PCR are shown above each exon. (B) Agarose gel analysis of RT-PCR products using primer pairs as indicated above the lanes. (+) and (-) indicate RT+ and RT- fetal ovary cDNA template. Size marker is the 100bp ladder (Promega) where the 500bp band is more intense. The arrow indicates the position of the weak band identified with the 2Fa + 3Rb primer pair. (C) Structure of the mouse *Nobox* locus for comparison with the human sequences.

Examination of GenBank and ENSEMBL database entries for the human *NOBOX* gene produced a number of putative transcripts identified *in silico* (Fig. 3A) but experimental evidence for any of them across all exons is lacking. Starting with the exons known to be expressed in adult ovarian follicles [41], we utilised RT-PCR with a number of their published primer pairs (Table 1, middle section) to confirm expression of these exons in the fetal ovary and then extended this analysis to test for the expression of the other putative exons identified more recently *in silico* using primers specific to those regions (Table 1, lower section).

Expression in human fetal ovary of the 5 exons identified previously in adult ovary (therein labelled exons 3 to 7 and indicated in black in Fig. 3A) [41] was confirmed (Fig. 3B gels 1 and 2; product sizes of 291bp, 773bp and 328bp respectively). Also in common with adult follicles [41], we did not detect the alternative splice product bands of 387bp, 869bp and 424bp respectively (Fig. 3B gels 1 and 2; shown in white in Fig. 3A and predicted in ENST00000467773) that would have been expected if the alternative splice product of their exon 4, adding a 32 amino acid extension to the homeodomain [42] was present.

RT-PCR with primers 7F + 8R and 7F + 9R indicated that the two most 3’ exons predicted for each transcript but not known at the time of the previous report [41] are present in *NOBOX* transcripts from human fetal ovary (Fig. 3B, gel 3, lanes 1–4; product sizes 522bp and 823bp).

**Fig 4:**
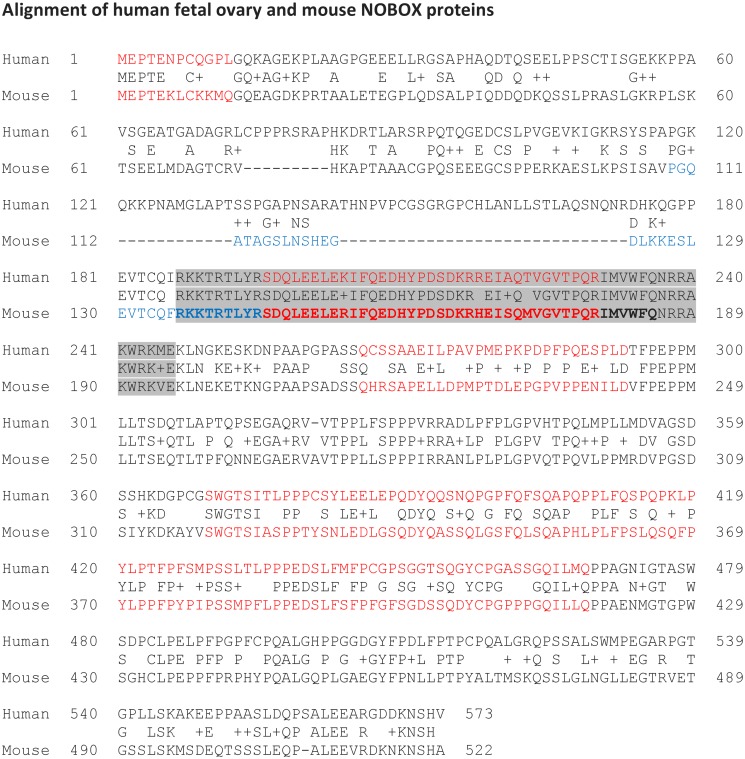
Protein sequence of the new human NOBOX isoform aligned with mouse Nobox. Human NOBOX protein (NP_001073882.2) and mouse Nobox protein (NP_570939.1) sequences are aligned. Alternating exons are coloured red and black, with the exon that is split in mouse coloured blue. The homeodomain is shaded grey.

Analysis of further combinations of primers yielded no other products (Fig. 3B, gel 3, lanes 6–11 and gel 4, lanes 2–5) or no product of the predicted size (183bp) but a low level of a larger product (approximately 454bp; Fig. 3B, gel 4, lanes 6–9)) which suggests retention of the 271bp intron between the 2 putative exons, compatible with a small amount of a partially processed transcript. While it is possible that our failure to detect products with these upstream primer sets is a result of at least one primer in each reaction being unsuitable for PCR, it seems unlikely given that several combinations have been used. A positive control reaction would be able to confirm this but since there is no existing *in vivo* evidence for any of these exons in fetal or adult ovary it is difficult to know how such a positive control could be derived. Thus, the three most 5’ exons of 85, 125 and 82nt from ENSEMBL transcripts ENST00000467773 and ENST00000483238 or the current Refseq transcript (NM_001080413.3) do not appear to be expressed in human fetal ovary. However primers 2Fc + 3Rb yielded a product of 139bp (Fig. 3B, gel 4, lanes 10–13), which together with the above results is consistent with the ENST00000223140 transcript being expressed in its entirety in human fetal ovary. Alignment of this gene structure with that of mouse *Nobox* (Fig. 3C) reveals considerable similarity in gene structure, in the absence of human 5’ and 3’UTRs, with the main difference being that the 552bp human exon is divided into 2 shorter exons in the mouse. Alignment of the predicted human and mouse proteins (Fig. 4) shows good conservation across most of the length of the protein (51.8% identity, 61.4% similarity) with the homology in the homeodomain being highest (87.5% identity). Thus we believe that we have identified the correct NOBOX coding sequence for human fetal ovary with the 3 most 5’ exons of the previously proposed sequence not present.

### Ectopic expression of NOBOX drives expression of target promoters through the NBE

**Fig 5:**
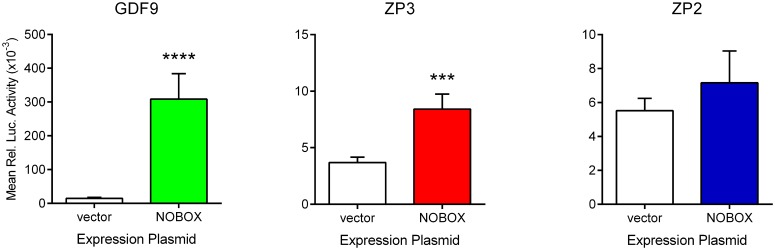
Expression of human NOBOX enhances expression from promoters containing NBEs in luciferase assays. Luciferase assays were performed on HEK293 cells transfected with a selection of promoter-luciferase plasmids containing (*GDF9* and *ZP3*) or lacking (*ZP2*) a putative NOBOX Binding Element (NBE) in combination with either NOBOX expression plasmid or empty vector. Data represent the mean (±sem) activity from at least 5 separate experiments. Statistically significant differences are denoted by asterisks.

Luciferase assays have previously been utilised to show that the proximal and distal NBEs present in the human *GDF9* promoter confer activation by co-expression with *NOBOX* expression plasmids [25]. However, the human NOBOX protein sequences used in previous experiments are derived from *in silico* derived transcripts for which we cannot demonstrate expression in the human fetal ovary. The sequence of the open reading frame derived from ENST00000223140, corresponding to the expressed fetal transcript, was codon optimised, synthetically manufactured (Origene Technologies Inc., Rockville, MD, USA; clone CW200571) and cloned into an expression vector (pCMV6-Entry). Co-transfection of HEK293 cells with a *GDF9* promoter—luciferase construct (pGL3-hGDF9) with the new *NOBOX* expression plasmid increased expression of luciferase relative to the β-galactosidase internal control by 21.2 +/- 4.0-fold compared to empty expression vector (Fig. 5; p<0.0001). Human *ZP3* and *ZP2* promoter luciferase constructs (positive and negative controls as we identified a putative NOBOX Binding Element (NBE) in the *ZP3* promoter 894bp upstream of the ATG start codon while there is none detectable within 2kb of the *ZP2* transcription start site) yielded a 2.25+/-0.16—fold increase in luciferase activity relative to empty vector for *ZP3* (Fig. 5; p = 0.0003) while there was no significant change in *ZP2* promoter activity (Fig. 5; p = 0.2).

## Discussion

These studies demonstrate that *GDF9* expression increases dramatically across gestation and is confined to small clusters of oocytes that also express activin βA, with little GDF9 present by the time oocytes form into primordial follicles. GDF9 expressing oocytes were significantly larger than those expressing DAZL, consistent with being at a more advanced stage of meiotic prophase I, but similar to those expressing BOLL [10]. It would therefore appear that during oogenesis in humans, oocytes go through a dynamic and very sharply demarcated sequence of changes in expression of various key regulators including activin βA, GDF9, DAZL and BOLL. These are likely to be of functional significance in determining selective germ cell survival at this critical stage in ovarian development.

Similarly to *GDF9*, mRNA expression of its known regulator *NOBOX* increased markedly during the same developmental period, confirming and extending previous data [34] and suggesting that it could play a role in the up-regulation of *GDF9* transcription at this stage. While human *NOBOX* was identified several years ago [41] and some *in vitro* analysis with human NOBOX on putative transcriptional targets has been performed [25], a proportion of the human *NOBOX* mRNA structure has only been derived *in silico* by exon prediction without experimental confirmation. We have identified a single, novel transcript and coding sequence that more closely resembles mouse *Nobox* than the *in silico*-derived isoforms [25] for which the only experimental evidence is the ability of derived recombinant products to bind and activate the GDF9 promoter *in vitro*. This novel transcript is also able to bind NBEs in the *GDF9* and *ZP3* promoters to *trans*-activate them.

Whether other *NOBOX* transcripts are expressed in adult ovary remains to be determined but it is interesting that only some of the *NOBOX* mutations identified in a cohort of women with POI and shown to affect NOBOX function *in vitro* [25] are encoded in the fetal transcript. This indicates that some identified mutations in NOBOX could result in loss of oocytes even before primordial follicle formation, whereas others, if they are expressed at later developmental stages, may result in POI at later ages, perhaps contributing to the variation in age of onset of the condition: further studies are required to determine NOBOX transcript structure in later fetal and postnatal life.

GDF9 signals through SMADs 2 and 3, previously localised to the somatic cells adjacent to germ cells in the human fetal ovary [12]. It was striking that both GDF9 and activin βA, which also signals through SMADs 2 and 3, are found in the same subset of oocytes in the human fetal ovary: signalling specificity is conferred by selective affinity for the Type I receptors ALK5 and ALK4 respectively [11]. In addition to canonical TGFβ superfamily signalling through Smad2 regulation of gene expression in *Drosophila melanogaster*, it has been shown that Smad2 can also bind the Activin subfamily receptor *baboon* during imaginal disc development to repress its activity non-canonically [43]. If a similar non-canonical activity of SMAD2 or SMAD3 on ALK4 in the human exists, potentially GDF9 activation of SMAD2/3 via ALK5 may have an inhibitory effect on ALK4 and thus activin signalling. While we have demonstrated the expression of BMP15 in the fetal ovary at the mRNA level, it was not possible to localise BMP15 protein to explore whether, as in later stages of oocyte maturation [44], GDF9 and BMP15 might be co-expressed. A number of other examples exist where TGFβ family members signalling through SMAD2/3 and those signalling through SMAD1/5/8 (*ie* the BMPs) co-exist and are even mutually dependent for correct developmental control [45].

The effect of activin βA on germ cell proliferation and survival is indirect, via effects on adjacent pre-granulosa cells [12,13]. We have proposed a model in which the suppression of KITL in adjacent pre-granulosa cells by germ cell-derived activin βA delays germ cell cyst breakdown and primordial follicle formation [14]. While GDF9 can also suppress expression of both KITL mRNA isoforms in pre-antral and mural granulosa cells in mice [46], opposite effects have been reported in other species and stages of follicle development [46,47]. While there is no direct evidence for GDF9 regulation of KITL expression in the human fetal ovary, it is possible that a balance between activin inhibition and GDF9 induction of KITL may determine the developmental progression and/or survival of individual oocytes as they progress towards follicle formation by regulating nest breakdown and thus the timing of primordial follicle formation. The selective regulation by NOBOX of GDF9 but not activin βA expression may contribute to this balance. Consistent with this, germ cell nest breakdown is compromised in *Nobox*-deficient mouse ovary [27] with major consequences for subsequent oocyte survival.

These data therefore demonstrate the expression of GDF9 by human oocytes prior to follicle formation and support the role of NOBOX as a master-regulator of germ cell fate [24] in the human fetal ovary as in the mouse, potentially acting as a determinant of the balance between GDF9 and activin βA signalling between germ cells and somatic cells at the critical time of follicle formation.

## Supporting Information

S1 Fig GDF9 Antibody Staining Controls. (A) GDF9 positive germ cells are present in a small number of germ cells in clusters in 16 week human fetal ovary. No GDF9 staining is present in 14 week fetal ovary (B) or 18 week human fetal testis (C) but the oocyte cytoplasm of both primordial and growing follicles in adult marmoset ovary is stained specifically with GDF9 antibody (D) and not normal goat IgG (E). Scale bars are as indicated.
(TIF)
