# Supplementary figures and images for: GDF9 is Transiently Expressed in Oocytes before Follicle Formation in the Human Fetal Ovary and is Regulated by a Novel NOBOX Transcript
Source: PLoS One. 2015 Mar 19;10(3):e0119819. doi: 10.1371/journal.pone.0119819 (PMC4366263; doi:10.1371/journal.pone.0119819)

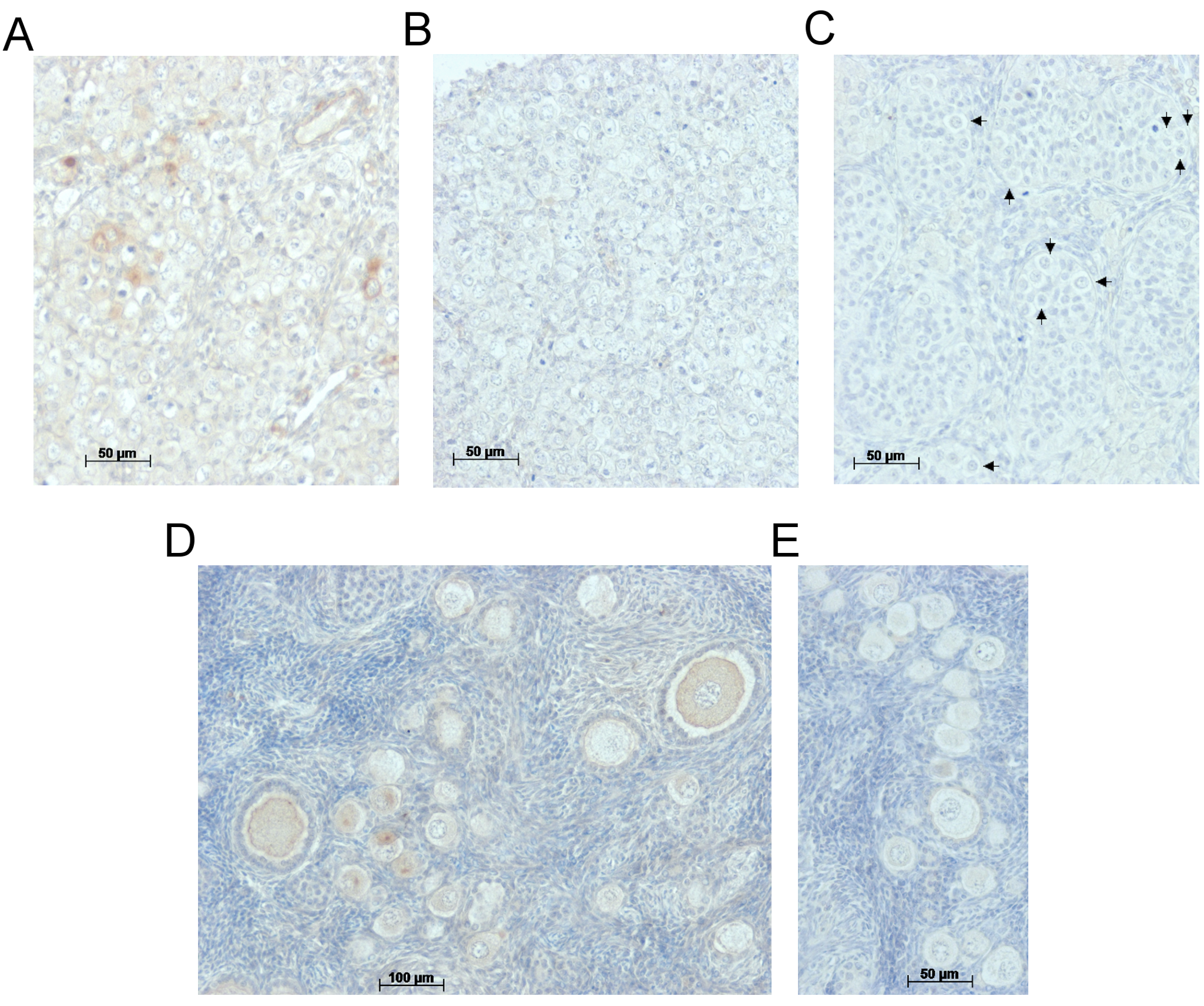

Supplement: S1 Fig — (A) GDF9 positive germ cells are present in a small number of germ cells in clusters in 16 week human fetal ovary. No GDF9 staining is present in 14 week fetal ovary (B) or 18 week human fetal testis (C) but the oocyte cytoplasm of both primordial and growing follicles in adult marmoset ovary is stained specifically with GDF9 antibody (D) and not normal goat IgG (E). Scale bars are as indicated. (TIF) [file pone.0119819.s001.tif]
